# Maternal dietary zinc supplementation enhances the epigenetic-activated antioxidant ability of chick embryos from maternal normal and high temperatures

**DOI:** 10.18632/oncotarget.15057

**Published:** 2017-02-03

**Authors:** Yongwen Zhu, Xiudong Liao, Lin Lu, Wenxiang Li, Liyang Zhang, Cheng Ji, Xi Lin, Hsiao-Ching Liu, Jack Odle, Xugang Luo

**Affiliations:** ^1^ Mineral Nutrition Research Division, Institute of Animal Science, Chinese Academy of Agricultural Sciences, Beijing 100193, P. R. China; ^2^ College of Animal Science, South China Agricultural University, Guangzhou 510000, P. R. China; ^3^ College of Animal Science and Technology, China Agricultural University, Beijing 100193, P. R. China; ^4^ Department of Animal Science, North Carolina State University, Raleigh, NC 27695, USA

**Keywords:** epigenetics, maternal hyperthermia, zinc, metallothionein, chick embryo

## Abstract

The role of maternal dietary zinc supplementation in protecting the embryos from maternal hyperthermia-induced negative effects via epigenetic mechanisms was examined using an avian model (*Gallus gallus*). Broiler breeder hens were exposed to two maternal temperatures (21°C and 32°C) × three maternal dietary zinc treatments (zinc-unsupplemented control diet, the control diet + 110 mg zinc/kg inorganic or organic zinc) for 8 weeks. Maternal hyperthermia increased the embryonic mortality and induced oxidative damage evidenced by the elevated mRNA expressions of heat shock protein genes. Maternal dietary zinc deficiency damaged the embryonic development associated with the global DNA hypomethylation and histone 3 lysine 9 hyperacetylation in the embryonic liver. Supplementation of zinc in maternal diets effectively eliminated the embryonic mortality induced by maternal hyperthermia and enhanced antioxidant ability with the increased mRNA and protein expressions of *metallothionein IV* in the embryonic liver. The increased *metallothionein IV* mRNA expression was due to the reduced DNA methylation and increased histone 3 lysine 9 acetylation of the *metallothionein IV* promoter regardless of zinc source. These data demonstrate that maternal dietary zinc addition as an epigenetic modifier could protect the offspring embryonic development against maternal heat stress via enhancing the epigenetic-activated antioxidant ability.

## INTRODUCTION

Maternal heat stress impairs the fetal and embryonic development in mammalian and avian species [1, 2]. Maternal hyperthermia induces the embryonic oxidative stress resulting from the excessive reactive oxygen species (ROS) in mammalian species [3, 4]. However, few studies have been performed to confirm this effect in avian species. Oxidative damage generated by maternal environmental stresses also can induce the aberrant epigenetic patterns [5]. Lower levels of global DNA methylation and histone acetylation from maternal stress contributed to the abnormal development and embryo death in mice and human [6, 7].

Zinc (Zn) deficiency resulted in the abnormal embryonic development and poor performing offspring in both hens [8] and mice [9]. The disruption of embryonic development due to Zn deficiency was associated with epigenetic defects such as the reduced methylation levels of DNA and histones, and these impairments were restored by dietary methyl donor supplementation [9]. Zinc as an antioxidant supplement can protect cells against oxidative damage from various environmental stimuli including heat stress [10, 11]. We have demonstrated that dietary supplementation with Zn increased mRNA and protein expressions of tissue metallothionein (MT) as free radical scavengers in broilers [12–14]. A moderately chelated organic Zn source was the most effective in augmenting tissue *MT* mRNA expression [13, 14]. *In vivo*, prenatal Zn deficiency increased *MT2* promoter DNA methylation and histone acetylation levels in liver of offspring mice [15]. *In vitro*, it is proved that *MT* gene mRNA expression was suppressed by DNA hypermethylation of its promoter region in cancer cells [16–19]. Therefore, the hypothesis of the present study was to investigate whether maternal dietary supplementations with different Zn sources could protect offspring chick embryos against the maternal hyperthermia-induced oxidative damage via epigenetic activation of *MT4* expression.

## RESULTS

### Embryonic development

**Figure 1:**
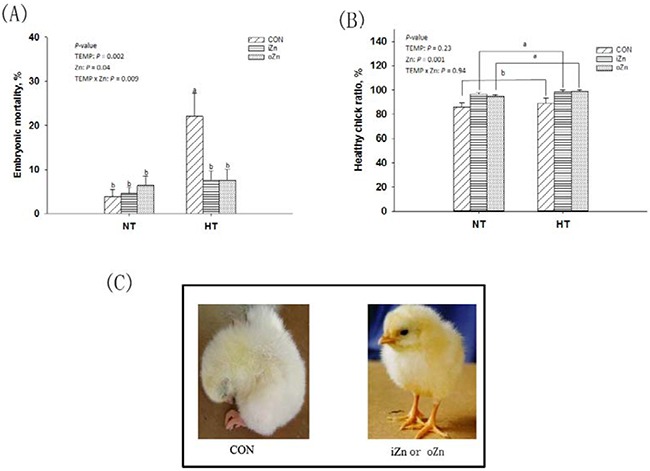
Effects of maternal environmental temperature and dietary Zn on the embryonic development **A**. Embryonic mortality was expressed as percentages of dead embryos in the total number of fertile eggs. **B**. Healthy chick ratio expressed as percentages of healthy chicks in the total number of hatched birds. **C**. The healthy and weak hatched chicks were presented. Based on the 2-way ANOVA analyses, lacking common letters (a or b, significant differences at *P* < 0.05) over a bar as determined by a main effect of maternal dietary Zn (*n* = 12) or their interaction (*n* = 6). All values are expressed as means ± SE.

Maternal temperature (TEMP) affected (*P* = 0.002) the embryonic mortality (Figure 1A), but had no effect (*P* > 0.22) on healthy chick ratio (Figure 1B). Maternal dietary Zn affected (*P* = 0.001) healthy chick ratio. There was an interaction (*P* = 0.009) in the embryonic mortality between maternal TEMP and dietary Zn, but no interaction (*P* > 0.90) was observed in the healthy chick ratio. Compared to maternal normal temperature (NT), maternal high temperature (HT) increased (*P* < 0.004) the embryonic mortality by 2.5-fold. Maternal dietary supplementation with either inorganic Zn (iZn) or organic Zn (oZn) had 11% higher (*P* < 0.05) healthy chick ratio than the control group (CON). Under NT, the embryonic mortality was not affected (*P* > 0.46) by maternal dietary Zn, while under HT, compared with CON, maternal dietary Zn supplementation decreased (*P* < 0.05) the embryonic mortalities to the similar level in the NT groups, but no difference (*P* > 0.72) was observed between the two Zn sources.

### Zn contents and antioxidant indices in the embryonic liver

**Figure 2:**
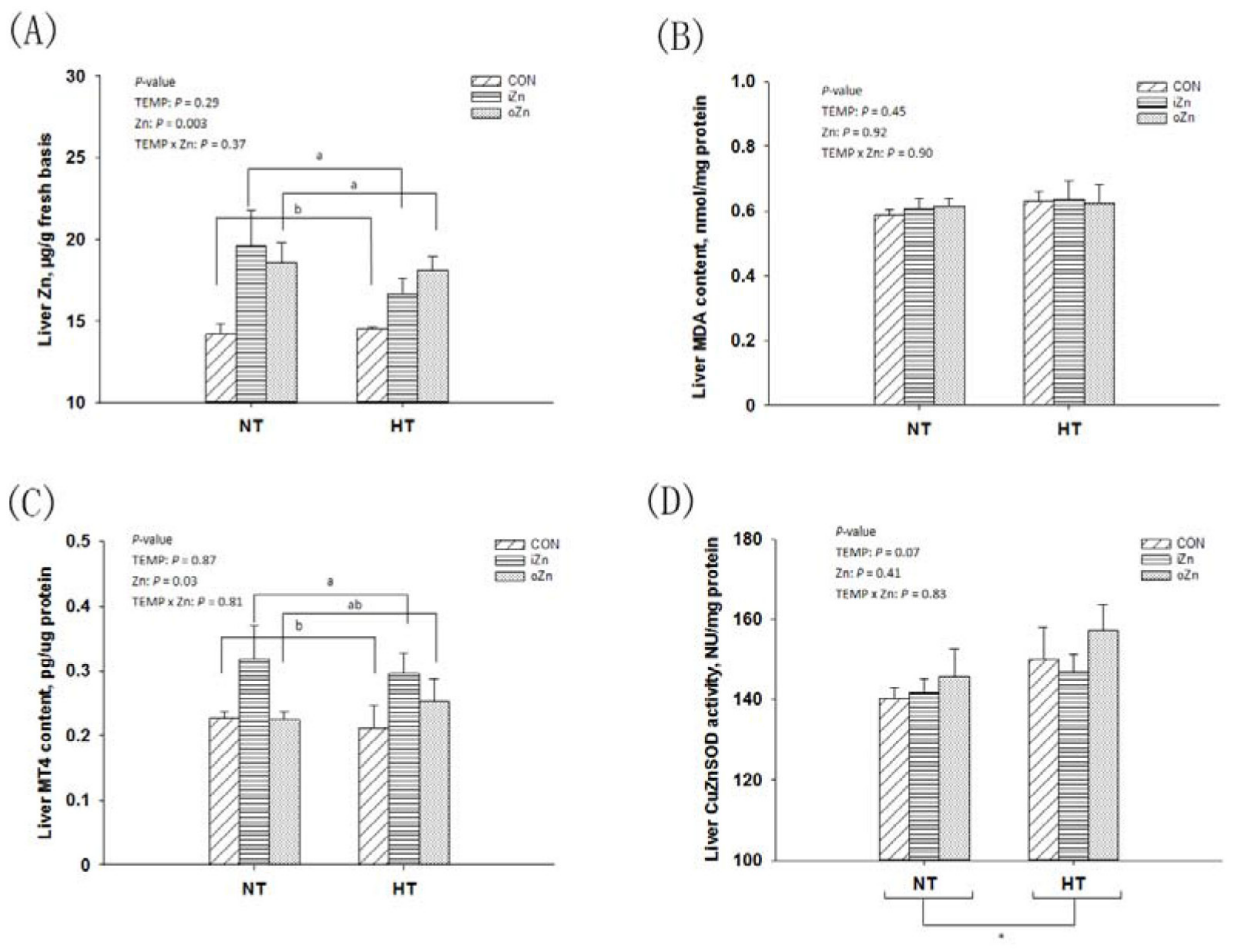
Effects of maternal environmental temperature and dietary Zn on Zn content and antioxidant ability in the embryonic liver The indices of Zn content **A**. MDA content **B**. MT4 content **C**. and CuZnSOD activity **D**. related with heat stress and dietary Zn in the embryonic liver were used to assess the antioxidant ability. Based on the 2-way ANOVA analyses, * (significant differences with a trend at 0.05 < *P* < 0.10) as determined by a main effect of maternal environmental temperature (*n* = 18); lacking common letters (a or b, significant differences at *P* <= 0.05) over a bar as determined by a main effect of maternal dietary Zn (*n* = 12). All values are expressed as means ± SE.

Liver as a main and sensitive target organ for Zn accumulation was also susceptible to heat stress [20]. Thus, focusing on the embryonic liver, we firstly measured the Zn content, malonaldehyde (MDA) and metallothionein IV (MT4) levels, and copper zinc superoxide dismutase (CuZnSOD) activity to determine whether maternal dietary supplementation with Zn could protect offspring embryos against maternal HT via enhancing antioxidant ability. Maternal TEMP had no effect (*P* > 0.28) on Zn content (Figure 2A), MDA (Figure 2B) and MT4 (Figure 2C) levels, but tended (*P* = 0.07) to affect CuZnSOD activity (Figure 2D) in the embryonic liver. Maternal dietary Zn affected (*P* < 0.04) Zn and MT4 contents, but had no effect (*P* > 0.40) on CuZnSOD activity and MDA content in the embryonic liver. There were no interactions (*P* > 0.36) in all of the above indices between maternal TEMP and dietary Zn. Compared to NT, HT tended (*P* = 0.07) to increase the liver CuZnSOD activity. Compared to the CON, maternal dietary Zn supplementation increased (*P <=* 0.05) Zn content in the embryonic liver with no difference (*P* > 0.30) between the two Zn sources. The MT4 level in the embryonic liver was higher (*P* = 0.004) in the iZn than in the CON, but no differences (*P* > 0.11) were observed between the oZn and CON or between the two Zn sources.

### Gene mRNA and protein expressions in the embryonic liver

**Figure 3:**
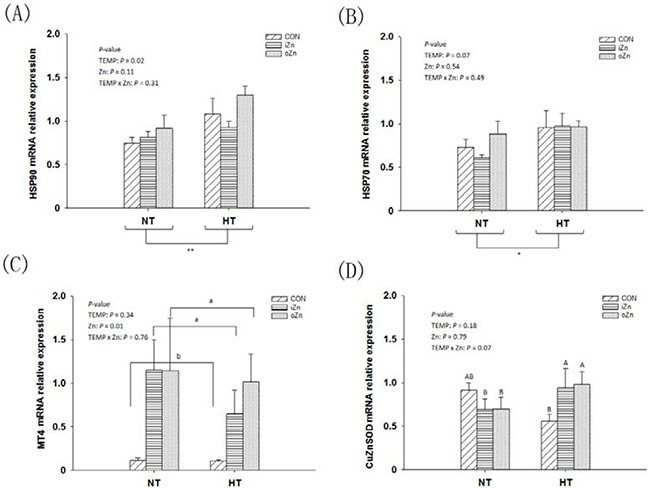
Effects of maternal environmental temperature and dietary Zn on gene mRNA expressions in the embryonic liver The mRNA expressions of *HSP90* **A**. *HSP70* **B**. *MT4* **C**. and *CuZnSOD* **D**. genes related with heat stress and antioxidant ability were determined in the embryonic liver. The geometric mean of internal references, *β-actin* and *GAPDH*, was used to normalize the expression of target genes. Based on the 2-way ANOVA analyses, ** (significant differences at *P* < 0.05) or *(significant differences with a trend at 0.05< *P* < 0.10) as determined by a main effect of maternal environmental temperature (*n* = 18); lacking common letters (a or b, significant differences at *P* < 0.05; A or B, significant differences with a trend at 0.05< *P* < 0.10) over a bar as determined by a main effect of maternal dietary Zn (*n* = 12) or their interaction (*n* = 6). All values are expressed as means ± SE.

**Figure 4:**
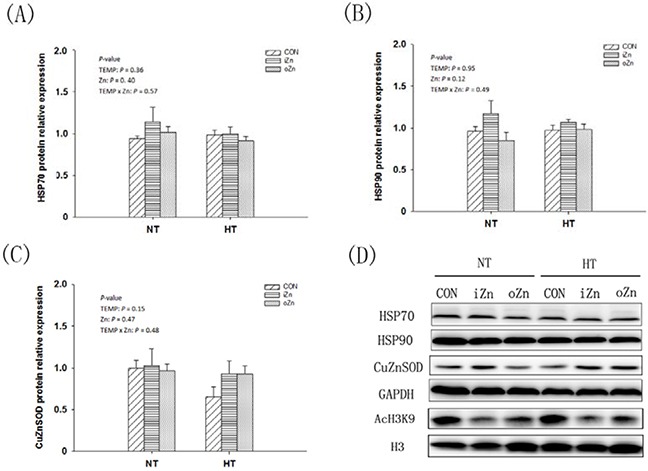
Effects of maternal environmental temperature and dietary Zn on protein expressions in the embryonic liver The data on protein expressions of HSP90 **A**. HSP70 **B**. and CuZnSOD **C**. in the embryonic liver were presented. **D**. Representative immunoblots of the indicated proteins were shown.

Compared to NT, maternal HT increased (*P* = 0.02) the liver heat shock protein 90 (*HSP90*) mRNA expression (Figure 3A) and tended to increase (*P* = 0.07) *HSP70* mRNA expression (Figure 3B), but had no effect (*P* = 0.34) on *MT4* mRNA expression in the embryonic liver (Figure 3C). Maternal dietary Zn affected (*P* = 0.01) the hepatic *MT4* mRNA expression, but had no effect (*P* > 0.10) on the *HSP90* and *HSP70* mRNA expressions. Maternal TEMP and dietary Zn tended (*P* = 0.07) to have an interaction on the *CuZnSOD* mRNA expression (Figure 3D), but had no effect (*P* > 0.30) on the rest indices. Maternal dietary Zn supplementation increased (*P* < 0.005) the *MT4* mRNA expression by 5-fold compared to the CON with difference (*P* > 0.62) between the two Zn sources. Under NT, no differences (*P* > 0.72) in the *CuZnSOD* mRNA expression were observed among maternal dietary Zn treatments, while under HT, maternal dietary Zn supplementation increased (*P* < 0.05) CuZnSOD mRNA expression compared with CON with no difference (*P* > 0.67) between the two Zn sources. Maternal TEMP, maternal dietary Zn and their interaction had no effects (*P* > 0.11) on the protein expressions of HSP70, HSP90 and CuZnSOD in the embryonic liver (Figure 4A–4C).

### Global and *MT4* promoter DNA methylation and H3K9 acetylation levels

**Figure 5:**
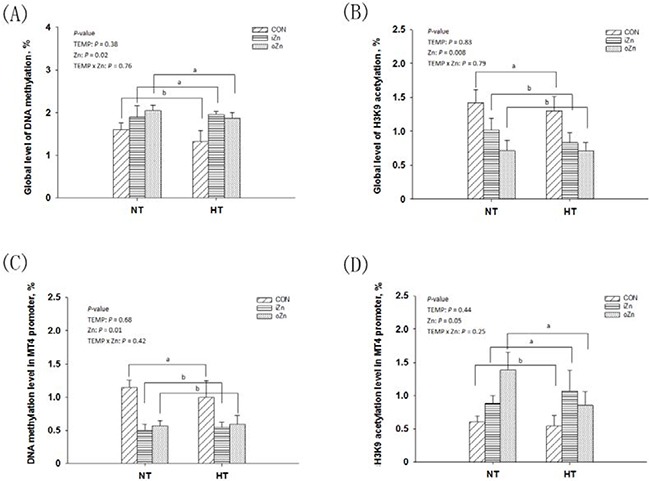
Effects of maternal environmental temperature and dietary Zn on DNA methylation and H3K9 acetylation in the embryonic liver The global levels of DNA methylation **A**. and H3K9 acetylation **B**. were determined using ELISA and Western-blot methods, respectively. The *MT4* promoter DNA methylation **C**. and H3K9 acetylation **D**. were determined using methylated DNA immunoprecipitation and chromatin immunoprecipitation methods, respectively. Based on the 2-way ANOVA analyses, lacking common letters (a or b, significant differences at *P* <= 0.05) over a bar as determined by a main effect of maternal dietary Zn (*n* = 12). All values are expressed as means ± SE.

The global level of DNA methylation and the relative degree of histone H3K9 acetylation after normalization to the total histone H3 level were measured in the embryonic liver (Figure 4D). Global DNA methylation (Figure 5A) and H3K9 acetylation (Figure 5B) levels were affected (*P* < 0.03) by maternal dietary Zn, but not (*P* > 0.37) by maternal TEMP or their interaction. Both the iZn and oZn had higher (*P* ≤ 0.05) global levels of DNA methylation and lower (*P* < 0.05) global levels of H3K9 acetylation than the CON with no difference (*P* > 0.38) between the two Zn sources. We further determined the DNA methylation (Figure 5C) and histone H3K9 acetylation (Figure 5D) levels of *MT4* promoter in the embryonic liver by a combination of immunoprecipitation (IP) and RT-PCR. The DNA methylation and H3K9 acetylation levels of the liver *MT4* promoter were affected (*P* ≤ 0.05) by maternal dietary Zn, but not (*P* > 0.24) by maternal TEMP or their interaction. Both the iZn and oZn had lower (*P* < 0.02) levels of DNA methylation and higher (*P* < 0.05) levels of H3K9 acetylation in the *MT4* promoter than the CON with no differences (*P* > 0.47) between the two Zn sources.

## DISCUSSION

As reported previously in mammals [3, 4], maternal heat stress increased embryonic mortality in laying hens in this study. The embryonic death from maternal hyperthermia was partially ascribed to the high susceptibility of embryos to oxidative stress in bovine [3] and porcine [4] species. The excessive ROS from oxidative stress induced the damages of lipid, protein and DNA and then arrested development of embryos [21, 22, 23]. However, lipid peroxidation was not observed in the livers of maternal heat-stressed embryos based on MDA content. It is implied that MDA content might be not a sensitive biomarker for reflecting oxidative damage of chick embryos during incubation. Additionally, ROS could act as second messengers by activating key transcription factors to alter target gene expressions [24]. The ROS can activate the binding activity of HSF1 and then increase the mRNA expressions of *HSP70* and *HSP90* in rats [25]. The HSP accumulations have been used as sensitive redox biomarkers for potential oxidative damage [26, 27]. In the present study, the oxidative damage was induced in embryos from maternal heat stress based on the elevation of *HSP90* and *HSP70* mRNA expression levels in the embryonic liver. However, a lack of correlation between the mRNA and protein expressions of either HSP90 or HSP70 was observed in the liver of maternal heat-stressed embryos. It is probably due to the reduced translational efficiency of gene mRNA [28] and rate of protein synthesis [29] in response to heat stress.

In the present study, Zn deficiency resulted in less high-quality hatched chicks (Figure 1C) from hens fed a semi-purified diet containing 9.98 mg of Zn/kg of diet, which confirmed the results of previous studies [8, 30]. Maternal dietary Zn deficiency significantly decreased Zn accumulation in embryos, and subsequently led to the abnormal development and loss of the embryos. Maternal dietary supplementation with Zn eliminated the embryonic mortality induced by maternal heat stress. Maternal dietary Zn supplementation increased *CuZnSOD* mRNA and *MT4* mRNA and protein expressions levels functioning as free radical scavengers in the embryonic liver from maternal heat stress. Therefore, the embryos from hens fed the diets supplemented with Zn might benefit from the enhanced antioxidant ability to prevent oxidative damage and embryonic mortality induced by maternal hyperthermia. Except for oxidative damage, the embryonic development was also impaired by the alteration of epigenetic processes, such as DNA methylation and histone modifications [6, 7, 31]. These epigenetic changes can further block or silence expressions of imprinted genes during embryogensis [7]. The lower global levels of DNA methylation and histone acetylation could contribute to the abnormal embryonic development [6, 9]. Zinc is necessary for the structure and function of DNA methyltransferases, methyl-binding proteins and deacetylases [32]. Maternal Zn-deficient diet induced the global DNA hypomethylation and H3K9 hyperacetylation in the embryonic liver, which confirmed the previous results reported in the liver of rats [9, 33, 34]. The reduced DNA methylation levels due to maternal Zn deficiency might be associated with the lower DNA methyltransferases activity and methyl group turnover rate [9, 34]. The abnormal epigenetic pattern was considered as one of reasons for the disruption of the embryonic development and embryo death. Gene expressions can be suppressed by DNA hypermethylation in CpG islands of the gene promoter region involved in a transcriptional repression mechanism [35]. In the current study, maternal Zn-deficient diet led to DNA hypermethylation of *MT4* promoter and inhibited *MT4* mRNA expression in the embryonic liver. The suppression of *MT* gene mRNA expression due to the higher levels of DNA methylation has been proven in various cancer cells [16–19]. In addition, the aberrant H3K9 hypomethylation accompanied by DNA hypermethylation was observed in the same region of the *MT4* gene. Histone deacetylation was associated with gene silencing via alterations in the structure of the nucleosomes or activation of RNA polymerase and other transcription coactivator binding [36, 37].

This study revealed that maternal hyperthermia increased the embryonic mortality and induced oxidative damage evidenced by the elevated mRNA expressions of *HSP90* and *HSP70* in the embryonic liver. Maternal dietary Zn deficiency impaired the embryonic development associated with the global DNA hypomethylation and H3K9 hyperacetylation in the embryonic liver. Maternal dietary supplementation with Zn enhanced antioxidant ability with increased *MT4* mRNA and protein expression levels against oxidative damage, and effectively eliminated the negative effect of maternal hyperthermia on the embryonic mortality. The *MT4* mRNA expression was increased by the reduced DNA methylation and the increased H3K9 acetylation of the *MT4* promoter. It is suggested that maternal dietary Zn addition as an epigenetic modifier could protect the embryonic development against maternal heat stress via enhancing the epigenetic activation of the antioxidant ability.

## MATERIALS AND METHODS

### Experimental design and treatments

A completely randomized design involving a 2 [maternal environmental temperatures, normal 21 ± 1°C (NT) and high 32 ± 1°C (HT)] × 3 [maternal dietary Zn treatments, a semi-purified diet without Zn supplementation as control basal diet (CON), the CON diet + 110 mg Zn/kg as either the inorganic (iZn) or organic Zn (oZn)] factorial arrangement of treatments was adopted. Thus, a total of 6 treatment groups (NT-CON, NT-iZn, NT-oZn, HT-CON, HT-iZn, and HT-oZn) were included. All eggs collected from the above 6 maternal treatments were incubated for 21 d.

### Animals and diets

This study with all experimental procedures was approved by Animal Welfare Committee of the Institute of Animal Science, Chinese Academy of Agricultural Sciences. A total of 144 18-wk-old female broiler breeders (Arbor Acres, Huadu Broiler Company, Beijing, China) were randomly allotted to 1 of 6 treatments with 6 replicates (4 birds per replicate) for each treatment according to the experimental design. All broiler breeder hens were initially adapted from 18 to 29 wk of age using a conventional corn-soybean meal diet. The body Zn stores were deleted for all the birds from 30 to 32 wk of age using a corn starch-isolated soybean protein purified diet without Zn addition. After that, the birds in 3 dietary Zn treatments from the NT group were housed at 21 ± 1°C, while those in 3 dietary Zn treatments from the HT group were reared at 32 ± 1°C for the experimental period from 33 to 42 wk of age. All hens were provided free access to tap water and the experimental diets. Semen was collected from male broiler breeders from 27 to 29 wk of age and mixed, and the volume and numbers of semen and sperm motility were determined before insemination. Artificial insemination was performed for twice a week.

The basal diets for the depletion period (corn starch-isolated soybean protein purified diet) and experimental period (corn-corn starch-isolated soybean protein semi-purified diet) were formulated to meet or exceed the requirements for laying broiler breeders, except for Zn (Table 1). According to the experimental design, inorganic Zn sulfate (reagent grade, ZnSO_4_·7H_2_O) and the Zn proteinate with a moderate chelation strength (feed grade, Q_f_ = 30.7) [14] were added to the basal diet based on the analysed Zn contents, respectively. The analysed Zn contents in diets are shown in Supplementary Table 1. To remove the effect of additional amino acids from the Zn proteinate, dietary methionine and lysine, as the first and second limited amino acids in poultry diets, were balanced to the same levels by adding synthetic DL-methionine and L-lysine·HCl to the control diet and diet supplemented with the inorganic Zn, according to supplemental amounts of methionine and lysine from the Zn proteinate source.

**Table 1:** Composition and nutrient levels of the basal purified and semi-purified diets for laying broiler breeders during the Zn depletion period and experimental period (as-fed basis)

Item	Zn depletion period	Experimental period
Ingredient, %		
Corn	-	35.20
Corn starch	67.48	35.20
Soy isolated protein	15.80	12.55
Soybean oil	1.50	1.40
Cellulose	3.00	3.00
CaCO_3_^1^	7.55	7.65
CaHPO_4_^1^	1.20	1.00
NaCl^1^	0.60	0.45
KH_2_PO_4_^1^	0.10	0.10
MgSO_4_·7H_2_O^1^	0.52	0.60
KCl^1^	0.10	0.30
DL-Met (98%)	0.45	0.40
Gly^1^	0.40	0.35
Micronutrients^2^	0.40	0.65
Corn starch + Zn additive, etc.^3^ additive^3^, et al.^3^	-	0.25
Nutrient composition, %		
ME^4^, MJ/kg	11.77	11.79
CP^5^	15.62	15.48
Lys^4^	0.87	0.78
Met^4^	0.57	0.56
Met + Cys^4^	0.65	0.70
Thr^4^	0.95	0.88
Gly^4^	0.92	0.55
Ca^5^	3.29	3.28
Nonphytate P^5^	0.46	0.47
Zn^5^, mg/kg	3.65	9.98

^1^Reagent grade.

^2^Provided per kilogram of diet for the Zn depletion period: vitamin A (retinyl acetate), 4500 IU; cholecalciferol, 450 IU; vitamin E (α-tocopherol acetate), 50.0 IU; menadione, 1.50 mg; thiamin, 13.4 mg; riboflavin, 15.0 mg; pyridoxine, 4.50 mg; cyanocobalamin, 0.02 mg; pantothenate, 18.0 mg; niacin 50.0 mg; folic acid 6.0 mg; biotin, 0.60 mg; choline (choline chloride), 1500 mg; Cu (CuSO_4_·5H_2_O), 10.0 mg; Fe (FeSO_4_·7H_2_O), 50.0 mg; Mn (MnSO_4_·H_2_O), 120 mg; I (KI), 1.20 mg; Se (Na_2_SeO_3_), 0.30 mg; Mo (NaMoO_4_·2H_2_O), 8.30 mg.

Provided per kilogram of diet for the experimental period: vitamin A (retinyl acetate), 11000 IU; cholecalciferol, 3500 IU; vitamin E (α-tocopherol acetate), 50.0 IU; menadione, 4.40 mg; thiamin, 6.60 mg; riboflavin, 12.0 mg; pyridoxine, 4.50 mg; cyanocobalamin, 0.02 mg; pantothenate, 15.5 mg; niacin 50.0 mg; folic acid 2.0 mg; biotin, 0.22 mg; choline (choline chloride), 2000 mg; Cu (CuSO_4_·5H_2_O), 10.0 mg; Fe (FeSO_4_·7H_2_O), 50.0 mg; Mn (MnSO_4_·H_2_O), 120 mg; I (KI), 1.20 mg; Se (Na_2_SeO_3_), 0.30 mg; Mo (NaMoO_4_·2H_2_O), 8.30 mg.

^3^Zinc additive, DL-Met, or L-Lys·HCl were added to diets by replacing an equal weight of corn starch.

^4^Calculated values.

^5^Analysed values based on triplicate determinations.

Eggs were collected from the last 3 wk of the experiment period. The eggs from each replicate of the all maternal treatments were kept on the same egg tray (6 trays totally) of one incubator (9TDJ-A, LanTianJiao Electronic Technology company, Beijing, China). The eggs were incubated at a temperature of 37.8°C and a relative humidity of 55 to 60% for 21 d. Eggs were candled at 7 d of incubation (E7) and E18.5 to identify nonviable embryos. All removed eggs on E7 and E18.5 were counted, opened, and visually evaluated also to determine the true embryonic mortality. On E21, the number of hatched healthy chicks was determined. Hatched chicks were classified as healthy birds if they could stand up and move freely, and breathed much more smoothly and developed better than weak birds [30]. Embryonic mortality was expressed as percentages of dead embryos in the total number of fertile eggs for each replicate per maternal treatment. Healthy chick ratio was expressed as percentages of healthy chicks in the total number of hatched birds for each replicate per treatment.

### Sample collections

On E18.5, 24 embryos (4 per replicate) from each treatment were killed by cervical dislocation. The livers from the embryos were immediately dissected and frozen in liquid nitrogen and then stored at -80°C for further analyses. Equal weight sub-samples of the livers from the 4 embryos in each replicate were pooled into one sample for analyses.

### Measurements of crude protein, calcium, zinc, nonphytate phosphorus

The crude protein and calcium contents in feed ingredients and diet samples and Zn contents in Zn proteinate, diets and the embryonic liver tissue were measured as described previously [14]. Dietary nonphytate phosphorus was determined as described previously [39].

### Determinations of antioxidant indices in the embryonic liver

As described previously [38], the homogenates of the embryonic liver samples were prepared to determine the total protein content, MDA and MT4 levels and CuZnSOD activity. The levels of total protein MDA and MT4 were determined using a BCA Protein Assay kit (Cat #23225, Pierce, Rockford, IL, USA), a commercial assay kit (Cat #A003-1, Nanjing Jiancheng Bioengineering Institute) and a Chicken MT4 ELISA Kit (Cat #CG3309, Waltham), respectively. The CuZnSOD activity was measured following the nitrite method [14]. All of the above indices were expressed as one unit per mg protein.

### Reverse transcription-qPCR assays

Total RNA was extracted from the embryonic liver tissues using Trizol reagent (Cat #15596018, Life Technologies) and then reverse-transcription was performed using QuantiTech Reverse Transcription Kit (Cat #205311, Qiagen) following the manufacturer's protocols with genomic DNA wiping off. The protocol of two-step PCR using ABI Fast PCR System was as follows: denaturation at 95°C for 2 min followed by 40 cycles at 95°C for 60 s, 60°C for 30 s, and 72°C for 30 s. The primer sequences are listed in Supplementary Table 2. The geometric mean of internal references, *β-actin* and glyceraldehyde 3-phosphate dehydrogenase (*GAPDH*), was used to normalize the expressions of the targeted genes. The 2^−ΔΔCt^ was used to calculate mRNA level of each target gene [38].

### Tissue preparations and western blotting assays

Total protein was extracted from frozen samples of the embryonic liver tissue as described previously [38]. The protein specimens (25 μg protein/lane) were separated on a 12% SDS-polyacrylamide gel and blotted on transferred to PVDF membranes (Cat #IPVH00010, Merck-Millipore). The blotted membranes were blocked with 5% skim milk for 1 h at room temperature and then incubated with the primary antibodies at 4°C overnight. The the secondary antibody was applied at room temperature for 1 h (Cat #CW0103A, ComWin Biotech, diluted 1:5,000). The antibodies used are listed in Supplementary Table 3. The signals were recorded and analysed using an ECL-plus detection system.

### Global DNA methylation quantification

The DNA was isolated from the embryonic liver samples using Lysis buffer, proteinase K (Cat #EO0491, Fermentas), and Rnase A (Cat #19101, Qiagen) and purified by phenol/chloroform extraction method. The methylation of the global DNA was detected using a MethylFlash Methylated DNA Quantification Kit (Cat #P1035, Epigentek) according to the manufacturer's instructions.

### Methylated DNA immunoprecipitation (MeDIP) analysis

The MeDIP analysis for gene promoter was performed as previously described [39] with some modifications. In brief, genomic DNA extracted from the embryonic liver samples was sonicated to produce small fragments ranging from 200 to 600 bp. The fragmented DNA (2 μg) was heat-denatured to produce single-stranded DNA and precleared with 20 μL of protein A/G agarose beads (50% slurry, Cat #sc-2003, Santa Cruz Biotechnology). A portion of DNA samples was saved as input (20 ng/μL) and the rest was immunoprecipitated with 5-methyl-cytidine antibodies and rotated at 4°C overnight (Cat #ab10805, Abcam). The immune complexes were captured with 30 μL of protein A/G agarose beads and then digested with 250 μL of digestion buffer containing proteinase K. Finally, the MeDIP DNA was purified. A small aliquot of MeDIP DNA and input DNA was used as the template for real-time PCR. We designed primers specific to proximal 5′ upstream promoter sequences of *MT4* (−1058 to -860 bp upstream from the transcription initiation site). The primer pair for MT4 produced a 165 bp amplicon to cover the promoter CpG island (106 bp) with 10 CpG sites (Supplementary Figure 1A). The relative changes in the extent of promoter methylation were determined by measuring the amount of promoter in immunoprecipitated DNA after normalization to the input DNA: % (Methylated DNA^IP/Input^) = 2^^[( Ct(MeDIP)-Ct(input)]^×100.

### Chromatin immunoprecipitation (ChIP) analysis

The preparation of sonicated chromatin from the embryonic liver tissue samples was performed according to previous publication [40]. The crude chromatin preparations were precleared with 20 μL of protein A/G agarose beads. Approximately 1/10 of the sample was collected as input control, while the remainder was immunoprecipitated with 1 μg of anti-acetyl H3K9, (Cat #12-371B, Millipore) overnight at 4°C with rotation. Immune complexes were captured with the protein A/G agarose beads. The DNA fragments from the immunoprecipitated complexes and input chromatin were released by reverse cross-linking at 65°C for 5 h and then were washed and purified. Purified ChIP DNA was used to amplify the gene promoter sequences of *MT4* by real-time PCR with the same specific primers as described in MeDIP analysis. ChIP with antibodies against AcH3K9, nonspecific IgG as a negative control (NC) and blank control (BC) was used to determine the fidelity of the ChIP protocol (Supplementary Figure 1B). The qPCR values were normalized to the values of the *GAPDH* promoter region. The fold enrichment of each target sequence was determined using the following formula [fold enrichment=2^−(Ct IpAcH3K9–Ct input)^] [40].

### Statistical analyses

Data were analysed by 2-way ANOVA using the general linear model procedure of the SAS 9.2 (SAS Institute Inc., Cary, NC), and the model included the main effects of maternal environmental temperature, maternal dietary Zn, and their interaction, and treatment comparisons for significant differences were tested by the LSD method. Embryonic mortality data were transformed to arcsine values before statistical analysis. Each replicate served as the experimental unit for all statistical analyses. Significant differences were set at *P ≤* 0.05 and at 0.05 < *P* < 0.10 for a trend.

## SUPPLEMENTARY MATERIALS FIGURES AND TABLES



## Abbreviations

ChIP: chromatin immunoprecipitation
CON: control group
CuZnSOD: copper zinc superoxide dismutase
HSP70 and HSP90: heat shock proteins 70 and 90
H3K9: histone 3 lysine 9
HT: high temperature
iZn: inorganic zinc
MDA: malonaldehyde
MeDIP: methylated DNA immunoprecipitation
MT4: metallothionein IV
NT: normal temperature
oZn: organic zinc
ROS: reactive oxygen species
TEMP: environmental temperature
Zinc: Zn.
