# Supplementary material for: Maternal dietary zinc supplementation enhances the epigenetic-activated antioxidant ability of chick embryos from maternal normal and high temperatures
Source: Oncotarget. 2017 Feb 3;8(12):19814–24. doi: 10.18632/oncotarget.15057 (PMC5386724; doi:10.18632/oncotarget.15057)
Supplement: Supplementary file 1 [file oncotarget-08-19814-s001.pdf]

Maternal dietary zinc supplementation enhances the epigenetic-activated antioxidant ability of chick embryos from maternal normal and high temperatures

SUPPLEMENTARY FIGURE AND TABLES

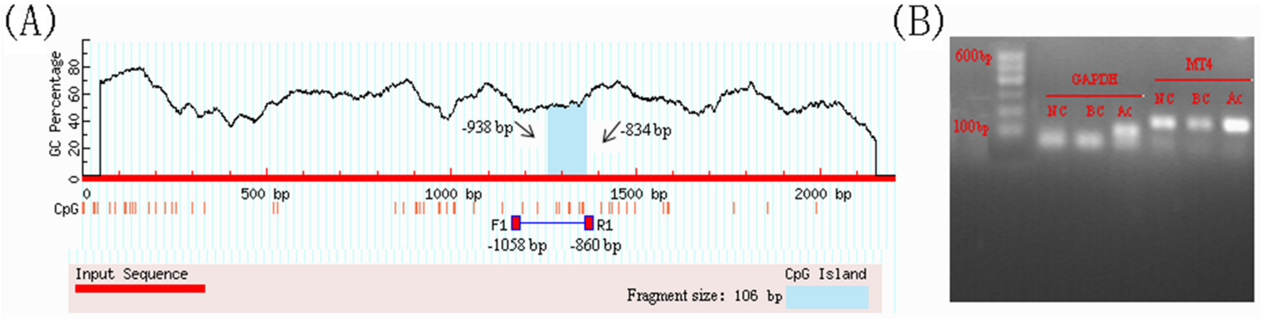

Supplementary Figure 1: The CpG-pattern rich regions in MT4 promoter and PCR results of ChIP for H3K9 acetylation. A. Schematic representations of MT4 and the CpG-pattern rich regions were presented. Red horizontal line indicates the input sequence as a total of 2200 bp. Blue vertical area shows the position of CpG sites within the -938 bp and -834 bp fragment. **B.** PCR results of ChIP protocol. ChIP with antibodies against AcH3K9 (**Ac**), rabbit IgG (**NC**) and blank control (**BC**) was used to determine the fidelity of the ChIP protocol in the embryonic liver.

Supplementary Table 1: Analyzed Zn contents in experimental diets[1]

Item	Dietary Zn treatments		
	CON	iZn	oZn
Added Zn, mg/kg	0	110	110
Analyzed dietary Zn, mg/kg[2]	9.98 ± 0.35	117 ± 3.7	116 ± 2.5

CON = the maternal Zn-unsupplemented basal diet; iZn = the maternal basal diet supplemented with 110 mg of Zn/kg of diet as $ZnSO_4 \cdot H_2O$; oZn = the maternal basal diet supplemented with 110 mg of Zn/kg of diet as Zn proteinate with a moderate chelation strength of 30.7 Q_f.

[1]The Zn contents are on an as-fed basis.

[2]Values are based on triplicate determinations and are expressed as means ± SD.

Supplementary Table 2: Summary of the primers used

Target gene	Gene bank accession no.	Product length, bp	Sequence (5'-3')	Applications
β-actin	NM_205518.1	95	F: ACCTGAGCGCAAGTACTCTGTCT'<br>R: CATCGTACTCCTGCTTGCTGAT	mRNA quantification
GAPDH	NM_204305.1	128	F: CTTTGGCATTGTGGAGGGTCR:<br>ACGCTGGGATGATGTTCTGG	
HSP70	JX_827854.1	144	F: CGTCAGTGCTGTGGACAAGAGTA<br>R: CCTATCTCTGTTGGCTTCATCCT	
HSP90	NM_001109785.1	108	F: GAGTTTGACTGACCCGAGCA<br>R: TCCCTATGCCGGTATCCACA	
CuZnSOD	NM_205064	119	F: CGCAGGTGCTCACTTTAATCC<br>R: CTATTTCTACTTCTGCCACTCCTCC	
MT4	NM_205275.1	163	F: AAGGGCTGTGTCTGCAAGGA<br>R:CTTCATCGGTATGGAAGGTACAAA	
MT4 promoter	ENSGALG00000011715	165	F: CGATCTGGCTGCAATCTACG<br>R: GAACCTGCCAGAACGCTAAG	MeDIP and ChIP
GAPDH promoter	ENSGALG00000014442	76	F: CCTGGTGGATCGTGTGTCG<br>R: CACACCACAAAGGTCAGGCG	ChIP

GAPDH, glyceraldehyde-3-phosphate dehydrogenase; *HSP70* and *HSP90*, heat shock proteins 70 and 90; *CuZnSOD,* copper zinc superoxide dismutase; *MT4,* metallothionein IV; MeDIP, methylated DNA immunoprecipitation; ChIP, chromatin immunoprecipitation.

Supplementary Table 3: Summary of the antibodies used

Antibodies	Molecular weight, KD	Host species	Source	Clonality	Catalogue no.	Dilution	Applications
GAPDH	37	Rabbit	Abcam	Polyclonal	Ab22555	1:5,000	Western blotting
HSP70	71	Rabbit	Abcam	Polyclonal	Ab69412	1:3,000	
HSP90	46	Rabbit	Abcam	Polyclonal	ab64182	1:3,000	
CuZnSOD	23	Rabbit	Santa	Polyclonal	sc-8637	1:1,000	
Histone H3	15	Mouse	Huaxingbio Science	Polyclonal	HX1850	1:5,000	
5-methylcytosine		Mouse	Abcam	Monoclonal	Ab10805		MeDIP
IgG		Rabbit	Millipore		12-371		ChIP
Anti-acetyl histone H3K9	17	Rabbit	Millipore	Polyclonal	ABE18		Western blotting and ChIP

GAPDH, glyceraldehyde-3-phosphate dehydrogenase; HSP70 and HSP90, heat shock proteins 70 and 90; CuZnSOD, copper zinc superoxide dismutase; DNMT3a and DNMT3b, DNA methyltransferases 3a and 3b; HDAC2, histone deacetylase2; KD, kilodalton; MeDIP, methylated DNA immunoprecipitation; ChIP, chromatin immunoprecipitation.
